# The OPTIMISE study protocol: a multicentre optimisation trial comparing continuous glucose monitoring, snacking habits, sleep extension and values-guided self-care interventions to improve glucose time-in-range in young people (13–20 years) with type 1 diabetes

**DOI:** 10.1007/s40200-022-01089-x

**Published:** 2022-08-11

**Authors:** Shelley Rose, Jillian J. Haszard, Barbara C. Galland, Esko J. Wiltshire, Martin I. de Bock, Carmel E. Smart, Miriama Ketu-McKenzie, Anna Campbell, Ruth Thomson, Craig A. Jefferies, Benjamin J. Wheeler, Sara E. Styles

**Affiliations:** 1 Department of Women’s and Children’s Health, University of Otago, Dunedin, New Zealand; 2 Department of Paediatrics & Child Health, University of Otago Wellington, Wellington, New Zealand; 3 Diabetes and Endocrinology Service, MidCentral District Health Board, Palmerston North, New Zealand; 4 Department of Human Nutrition, University of Otago, Dunedin, New Zealand; 5 Biostatistics Centre, University of Otago, Dunedin, New Zealand; 6 Paediatric Department, Capital and Coast District Health Board, Wellington, New Zealand; 7 Department of Paediatrics, University of Otago, Christchurch, New Zealand; 8 Paediatric Department, Canterbury District Health Board, Christchurch, New Zealand; 9 Hunter New England Local Health District, John Hunter Children’s Hospital, New Lambton Heights, NSW Australia; 10 School of Medicine and Public Health, University of Newcastle, Callaghan, NSW Australia; 11 Ngāti Tūwharetoa and Ngāti Raukawa (ki Horowhenua), Dunedin, New Zealand; 12 Paediatric Department, Southern District Health Board, Dunedin, New Zealand; 13 Dietetic and Nutrition Services, Southern District Health Board, Dunedin, New Zealand; 14 Paediatric Diabetes and Endocrinology Service, Starship Children’s Health, Auckland, New Zealand; 15 Department of Paediatrics, Liggins Institute, The University of Auckland, Auckland, New Zealand; 16 Department of Human Nutrition, University of Otago, PO Box 56, 9054 Dunedin, New Zealand

**Keywords:** Type 1 diabetes, Adolescents, Snacking, Sleep, Social values, Continuous glucose monitoring

## Abstract

**Purpose:**

The OPTIMISE study uses a Multiphase Optimisation Strategy (MOST) to identify the best combination of four interventions targeting key diabetes self-care behaviours for use in clinical practice to improve short-term glycaemic outcomes.

**Methods:**

This 4-week intervention trial will recruit 80 young people (aged 13–20 years) with type 1 diabetes ≥ 6 months duration), and pre-enrolment HbA1c ≥ 58 mmol/mol (7.5%) in the prior 6 months. Both main intervention and interaction effects will be estimated using a linear regression model with change in glucose time-in-range (TIR; 3.9–10.0 mmol/L) as the primary outcome. Participants will be randomised to one of 16 conditions in a factorial design using four intervention components: (1) real-time continuous glucose monitoring (CGM), (2) targeted snacking education, (3) individualised sleep extension, and (4) values-guided self-care goal setting. Baseline and post-intervention glucose TIR will be assessed with blinded CGM. Changes in self-care (snacking behaviours, sleep habits and duration, and psychosocial outcomes) will be assessed at baseline and post-intervention to determine if these interventions impacted behaviour change.

**Discussion:**

The study outcomes will enable the selection of effective and efficient intervention components that increase glucose TIR in young people who struggle to achieve targets for glycaemic control. The optimised intervention will be evaluated in a future randomised controlled trial and guide the planning of effective clinical interventions in adolescents and young adults living with type 1 diabetes.

**Trial registration:**

This trial was prospectively registered with the Australian New Zealand Clinical Trials Registry on 7 October 2020 (ACTRN12620001017910) and the World Health Organisation International Clinical Trails Registry Platform on 26 July 2020 (Universal Trial Number WHO U1111-1256-1248).

## Background

Type 1 diabetes (T1D) is the most common form of diabetes in young people [1]. Current guidelines recommend a target HbA1c < 53 mmol/mol (< 7.0%) [2] and interstitial glucose target of 70% time-in-range (TIR: 3.9–10.0 mmol/L) [3] to prevent long-term diabetes complications [4]. Despite advances in treatment and innovations in diabetes technology, glycaemic control deteriorates during the transition from childhood to young adulthood [5]. Systematic reviews of intervention trials to improve clinical and psychosocial outcomes in young people with type 1 diabetes suggest multi-component interventions may be more effective than single-component interventions among adolescents [6], although the evidence for young adults is limited [7].

A multiphase optimisation strategy (MOST) is a systematic method used to develop effective, robust, and scalable behavioural interventions [8]. MOST consists of three stages: (1) preparation, (2) optimisation and (3) evaluation of the optimised intervention in an RCT [8]. The present study focuses on optimisation, in which candidate intervention components that were carefully selected during the preparation phase will be evaluated against a clinically meaningful performance criterion. The combination of components that achieve the greatest improvement in TIR will comprise the ‘optimised’ intervention package. The effectiveness of the optimised intervention will then be evaluated in a future standard randomised controlled trial.

**Fig. 1:**
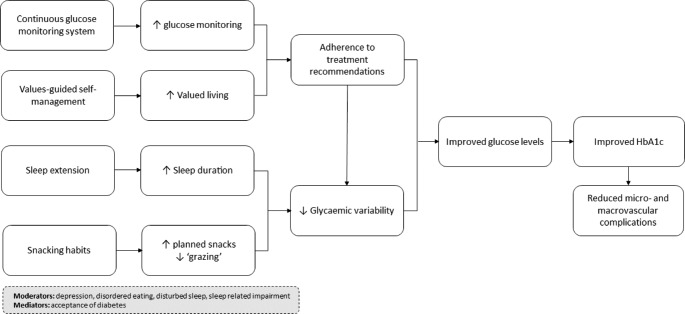
The OPTIMISE Study conceptual model

As part of the preparation phase, four candidate components were selected to target diabetes self-care behaviours that are important for young people to achieve glycaemic control targets. The conceptual model underlying the intervention is presented in Fig. 1.

### Continuous glucose monitoring

Glucose monitoring 6–10 times each day is recommended to inform treatment decisions and subsequently glycaemic control [9]. Adherence to multiple finger-prick tests per day is difficult [10]. Real-time continuous glucose monitoring (rtCGM) increases TIR and reduces HbA1c among adolescents and young adults [11]. A synergistic effect of glucose monitoring technology use with additional self-care support warrants exploration [12].

### Snacking habits

Skipping meals, increased or inconsistent snacking behaviours (or ‘grazing’), and insulin omission with snacks contribute to suboptimal glycaemic control [13]. Educating young people to take insulin boluses for snacks is associated with healthier glycaemic control [14]; however, no interventions have targeted incorporating planned snacks into a routine meal plan. This study will explore a pragmatic intervention to extend previous research on snacking.

### Sleep extension

Recent research highlights sleep as an important target for improving glycaemic control among youth with type 1 diabetes [15]. There is likely a bidirectional relationship between sleep and glycaemic control [16], with sleep disrupted by variability in timing due to night-time self-care, later bedtimes as adolescents mature, and early waking times dictated by school and work schedules. Sleep extension is feasible in youth (ages 10–16 yrs) [17] and warrants further investigation in older youth.

### Values-guided diabetes self-management

Values-guided diabetes self-management encourages personal choice, which is ideally suited to adolescence and young adulthood when individuals are becoming increasingly autonomous. Values-guided self-management is theorised to motivate youth to accept the negative aspects of their condition (e.g., pain, perceived stigma, undesirable glucose values) and adhere to treatment because doing so is acting in accordance with the person one wants to be or contributing to a more meaningful life instead of ‘the numbers’ (HbA1c targets) [18]. This approach can support self-management among adults with type 2 diabetes [19] and may improve quality of life among adolescents with a chronic condition [20].

This study aims to evaluate the effectiveness of four individual intervention components to (1) estimate the individual and synergistic effects of the four individual interventions on glucose TIR and determine which combination(s) offer the greatest improvements, (2) explore potential mediators and moderators of the efficacy of each of the four individual intervention components, and (3) provide evidence for an intervention or combination that optimises outcomes for youth with type 1 diabetes.

## Methods

### Study design

The proposed research is an optimisation trial using a 2^4^ factorial experimental study design (Table 1) [8]. This design is more economical compared to conducting four randomised controlled trials of the separate components because fewer participants are required to achieve the same power [8]. Using a correlation between measures of 0.6 and a standard deviation of 10 (based on previously published data) [12] a sample size of 80 is required. This sample size (5 participants in each of the 16 groups and 40 assigned to each component – see Table 1) gives 80% power at α = 0.05 level to detect an improvement of 5% TIR. Using effect coding, the interaction terms (which assess combinations of intervention components) are equally powered to the main effects.

**Table 1:** Experimental conditions in the OPTIMISE Study 2^4^ factorial experiment

Experimental condition	CGM	SLP	SNK	VAL	n
1	NO*	NO	NO	NO	5
2	NO	NO	NO	YES	5
3	NO	NO	YES	NO	5
4	NO	NO	YES	YES	5
5	NO	YES	NO	NO	5
6	NO	YES	NO	YES	5
7	NO	YES	YES	NO	5
8	NO	YES	YES	YES	5
9	YES	NO	NO	NO	5
10	YES	NO	NO	YES	5
11	YES	NO	YES	NO	5
12	YES	NO	YES	YES	5
13	YES	YES	NO	NO	5
14	YES	YES	NO	YES	5
15	YES	YES	YES	NO	5
16	YES	YES	YES	YES	5
Total	-	-	-	-	80

*NO means not allocated to the intervention: YES means allocated to the intervention

Abbreviations: CGM, continuous glucose monitoring; SLP, sleep extension; SNK, snacking intervention; VAL, values-guided self-management

## Study procedures

### Participant eligibility and recruitment

The research is being conducted across four academic centres (University of Otago – Dunedin, Christchurch, and Wellington campuses, and The University of Auckland) with established strong collaborations with District Health Boards (DHBs) covering approximately three-quarters of New Zealand (NZ)’s total population.

Participants will be invited to participate during a routine clinic visit by diabetes care providers affiliated with the academic centres and advertisements through NZ diabetes organisations’ social media accounts and websites. Eligibility criteria include: age 13–20 years, inclusive; type 1 diabetes for at least six months; mean HbA1c ≥ 58 mmol/mol (7.5%) in the prior six months. Exclusion criteria include: severe diabetes-related complications (e.g., nephropathy on treatment), severe depression requiring treatment, diagnosed sleep or eating disorders under active treatment; habitual sleep duration > 10 h; shift worker (works or has plans to work at least 3 h between midnight and 0500 h during the intervention period); current use of rtCGM technology (use of intermittently scanned CGM not excluded).

### Intervention component procedures

Each component will aim to facilitate behaviour change during a 4-week intervention phase using minimal resources. Components will be delivered 1:1 by trained research staff (in person or via Zoom). A full description of components is available in the supplemental file. Protocols were developed to standardise component delivery with careful consideration of the burden for youth allocated to the intervention and staff delivering it. A pragmatic approach guided component content and delivery to maximise the likelihood the optimised intervention will be acceptable and scalable across NZ.

### Glucose monitoring

The glucose monitoring component will use the Dexcom® G6 CGM system (Dexcom, Inc., San Diego, CA, USA) linked to a personal mobile device. The system collects glucose data every five minutes, is approved for making diabetes treatment decisions without confirmatory finger-prick tests and facilitates glucose data sharing. The CGM component also consists of personalised feedback on recent CGM data, goal setting, and a 2-week review of CGM data.

### Snacking habits

The snacking component consists of written, verbal, and visual information about healthful snacking, an action planning activity that if adopted is likely to improve snacking habits, and a 2-week review of the snacking goal identified in the action planning activity. In this research, a ‘snack’ is defined as a food or drink (not including water) consumed between main meals and does not include treatment for hypoglycaemia. The snacking component was developed by S.R., C.E.S., R.T. and S.E.S.

### Sleep extension

Participants allocated to the sleep extension component will be encouraged to go to bed one hour earlier than their usual bedtime on weekdays and weekends (as determined by their baseline self-reported ‘lights out’ time), while maintaining their usual wake-up time. Sleep hygiene education will be provided to support participants in achieving the goal. The sleep extension goal will be reviewed at the 2-week follow-up visit. The protocol was adapted from B.C.G.’s previous research.

### Values-guided diabetes self-management

The values-guided diabetes self-management component will comprise a 30–60 min psychoeducational session delivered 1:1 by a researcher (S.E.S.) who completed advanced workshops in Acceptance and Commitment Therapy (ACT) [21] and He Puna Whakaata (a values-based mātauranga Māori [Māori knowledge] programme for practitioners). The session will follow a protocol inspired by a brief values-informed healthy lifestyle intervention for young adults and the DNA-V (i.e., Discoverer, Noticer, Advisor and Values) model of behavioural interventions for youth [22], which focuses on a participant’s self-chosen diabetes-specific problem and an action planning activity that if adopted would be likely to improve the problem. A psychoeducational handout based on the DNA-V model will provide strategies for overcoming psychological barriers to self-management. The action plan will be reviewed at the 2-week follow-up visit. Expert review of this component was undertaken by clinical psychologists (A.C., M.K.) to ensure fidelity to the underpinning theoretical basis for the intervention and cultural appropriateness.

### Outcome assessments

Outcomes to be assessed are outlined in Table 2.

**Table 2:** Overview of assessments

Timepoint	Purpose	Measure*
Screening visit (Day − 14)	Demographics	Retrieved from medical records: Age, gender, ethnicity, education/occupation status, residential address
	Clinical characteristics	Retrieved from medical records: Height, weight, body mass index (BMI), BMI z-score, date of diagnosis, duration of diabetes, insulin regimen, total daily insulin dose, HbA1c (measured at point-of-care), severe diabetes-related complications and/or psychiatric comorbidities
	Depression score	Patient Health Questionnaire (PHQ):[27] The PHQ-A will be administered to adolescents aged 13–17 years and the PHQ-9 will be administered to young adults ages 18 + years; screen for presence and severity of depression over the previous 2 weeks; scores ≥ 15 suggest moderately severe to severe depression
	Disordered eating behaviour	Diabetes Eating Problem Survey-Revised (DEPS-R):[28] All participants asked to complete DEPS-R screen for disordered eating behaviour over the past month; scores ≥ 20 suggest disordered eating behaviour
Baseline visit (Day 0)	Review blinded CGM	Percent time-in-range (TIR: 3.9–10.0 mmol/L) prior over 14 days as measured by FreeStyle Libre Pro blinded CGM
	Review Actigraphy	Sleep duration objectively measured for 7 days (8 nights)
	Diabetes self-care	Self-Care Inventory-Revised (SCI-R):[23] Assess adherence to diabetes self-care recommendations over previous 30–60 days; 15 items on a 5-point Likert scale; higher scores indicate more optimal self-care behaviours
	Snacking habits	Complete questionnaires to assess the following over the previous 7 days: timing and frequency of planned snack foods and beverages consumed during the day and before bed; frequency of grazing episodes; frequency of intake of commonly consumed snack foods and beverages
	Sleep hygiene	Adolescent Sleep Hygiene Scale:[25] Assess sleep-facilitating and sleep-inhibiting practices over the last month. Higher scores indicate better sleep hygiene
	Sleep disturbance	Patient Reported Outcomes Measurement Information System (PROMIS)® Sleep-Related Impairment short form (v 1.0; 8a)[30] and the PROMIS Sleep Disturbance short form (v 1.0; 8a):[30] Assess sleep-related impairment and disturbance over the past 7 days; higher scores represent more of the concept being measured, e.g., worse sleep-related impairment
	Acceptance of diabetes	Diabetes Acceptance and Action Scale-Revised (DAAS-R):[29] Assess current acceptance of diabetes; higher scores indicate greater acceptance of diabetes
	Valued living	Valuing Questionnaire (VQ):[26] Self-reported alignment and interference with living consistently with one’s values over the past 7 days
Follow-up visit (Day 14)	Repeat self-care questionnaires	Self-Care Inventory-Revised (SCI-R);[23] snacking questionnaires; PROMIS Sleep-Related Impairment short form (v 1.0; 8a)[30] and Sleep Disturbance short form (v 1.0; 8a);[30] Adolescent Sleep Hygiene Scale;[25] Diabetes Acceptance and Action Scale-Revised (DAAS-R);[29] Valuing Questionnaire (VQ)[26]
Final visit (Day 28)	Review blinded CGM and Actigraphy Repeat self-care questionnaires	Percent time-in-range (TIR: 3.9–10.0 mmol/L) prior over 14 days as measured by FreeStyle Libre Pro blinded CGM Sleep duration objectively measured for 7 days (8 nights) As above

*All participants completed the same set of questionnaires. Questionnaires with an age-specific version were administered where relevant

Abbreviations: CGM Continuous Glucose Monitoring

### Primary outcome

The FreeStyle Libre Pro (Abbott Diabetes Care, Witney, Oxon, U.K.) blinded CGM system will be used to measure interstitial glucose data for 14 days and assess change in TIR.

### Secondary outcomes

Changes in self-care will be assessed in participants only (not parents) to determine if these interventions impacted behaviour change. The participant’s self-care data will be collected using electronic questionnaires administered via REDCap (Research Electronic Data Capture), a password protected secure web-based application hosted at the University of Otago.

### Diabetes self-management

The Self-Care Inventory-Revised (SCI-R) [23] is a validated measure of perceived adherence to diabetes self-care recommendations among youth with diabetes. Responses to 15 items are given on a 5-point Likert type scale from 1-Never to 5-Always, with higher scores indicating more optimal self-care behaviours.

### Snacking habits

A questionnaire developed by S.E.S., J.J.H., S.R., and C.E.S. to assess snacking behaviours over the previous seven days. Questions about the timing (between breakfast and lunch, between lunch and dinner, after dinner) and frequency (from never to most days) of planned snacks eaten as part of a routine meal plan are included, and self-reported caffeine and alcohol consumption before bed. Snacking behaviours during the school/workday and on weekends/during holidays will be reported separately and assessed for accuracy using the rtCGM traces. The frequency of grazing episodes (from none to 3 + per day) will be assessed using a question from a survey of adolescents and adults aged 15 + years [24]. A food frequency questionnaire will assess how often 13 categories of common snack foods (e.g., fruit, breakfast cereals, packaged snacks, rice/pasta/noodles) and beverages were consumed in the past seven days (from none to 3 + per day). The final questionnaire was modified based on their feedback. The snacking behaviours and snack foods questionnaires were pre-tested with a sample of young people with type 1 diabetes and modified based on their feedback.

### Sleep habits and duration

Self-reported usual ‘lights out’, wake-up time, and hours of sleep on weeknights and weekend nights over the past seven days will be used to confirm eligibility and tailor recommendations to participants in the sleep extension component.

The Adolescent Sleep Hygiene Scale is a 33-item questionnaire to assess sleep-facilitating and sleep-inhibiting practices, with good internal consistency (Cronbach α = 0.80) [25]. Participants report how often each sleep item occurred over the last month along a 6-point ordinal rating scale. Higher scores indicate better sleep hygiene.

Participants will wear an ActiGraph (ActiGraph© wGT3X-BT, LLC, Florida, U.S.A.) on the non-dominant wrist for seven days and eight nights (continuously), to obtain objective sleep data. ActiGraphs will be initialised using 15s epochs. Participants will complete a sleep diary over the same period to record unusual or disturbed sleep to assist in interpreting sleep data. The actigraphy data will be downloaded into ActiLife Software (version 6.0 or later) and analysed with an automated script developed in MATLAB® (MathWorks, Natick, MA, USA). The script uses a count-scaled algorithm to estimate sleep onset and offset for sleep and awakenings. Participants will be excluded from the analysis if fewer than three valid nights of wear are obtained.

### Valued living

The Valuing Questionnaire (VQ) [26] is a validated measure of how consistently an individual has been living with their self-determined values in the past week and is used to evaluate ACT interventions. The VQ contains 10-items rated from 0 (not at all) to 6 (completely true). Higher scores indicate more consistently living by one’s self-determined values.

### Moderators

Baseline depression will be assessed using the Patient Health Questionnaire (PHQ) [27]. The PHQ-A will be administered to adolescents aged 13–17 years and the PHQ-9 will be administered to young adults ages 18 + years; the 9-item questionnaires answered on a 4-point Likert type scale from 0 (not at all) to 3 (nearly every day). Scores ≥ 15 suggest moderately severe to severe depression.

Baseline disordered eating will be assessed with the Diabetes Eating Problem Survey-Revised (DEPS-R); this 16-item tool is used to assess general and diabetes-specific disordered eating behaviours including weight loss, food restriction, insulin misuse, and vomiting [28]. The DEPS-R is scored on a 6-point Likert scale ranging from never to always, with scores ≥ 20 indicating the tendency towards disordered eating behaviours.

### Mediators

The Diabetes Acceptance and Action Scale-Revised (DAAS-R) is a brief reliable and valid measure of acceptance of diabetes used in clinical and research settings among patients aged 16 years and older [29].

Both the Patient Reported Outcomes Measurement Information System (PROMIS)® Sleep-Related Impairment short form (v 1.0; 8a) [30] and the PROMIS Sleep Disturbance short form (v 1.0; 8a) [30] will be used to assess sleep-related impairment and disturbance, respectively, over the past seven days. Each questionnaire contains eight items, each item having five response options ranging in value from 1 to 5, with higher scores representing more of the concept being measured.

## Data collection procedure

### Demographic and clinical data

Demographic information will be self-reported via questionnaire or obtained from medical records including: age, gender, diabetes duration, height, weight, body mass index, insulin regimen total, self-identified ethnicity, employment or education status, living situation (e.g., 1- or 2-parent/caregiver household or flatting), address (to determine NZDep2018 score, a validated measure of socioeconomic status) [31]. Baseline HbA1c will be measured with a point-of-care device (DCA Vantage Analyzer, Siemens Healthcare Diagnostics, Ireland), linked directly to the Diabetes Control and Complications Trial method [certified through the National Glycohemoglobin Standardization Program (NGSP)].

### Study visits

**Fig. 2:**
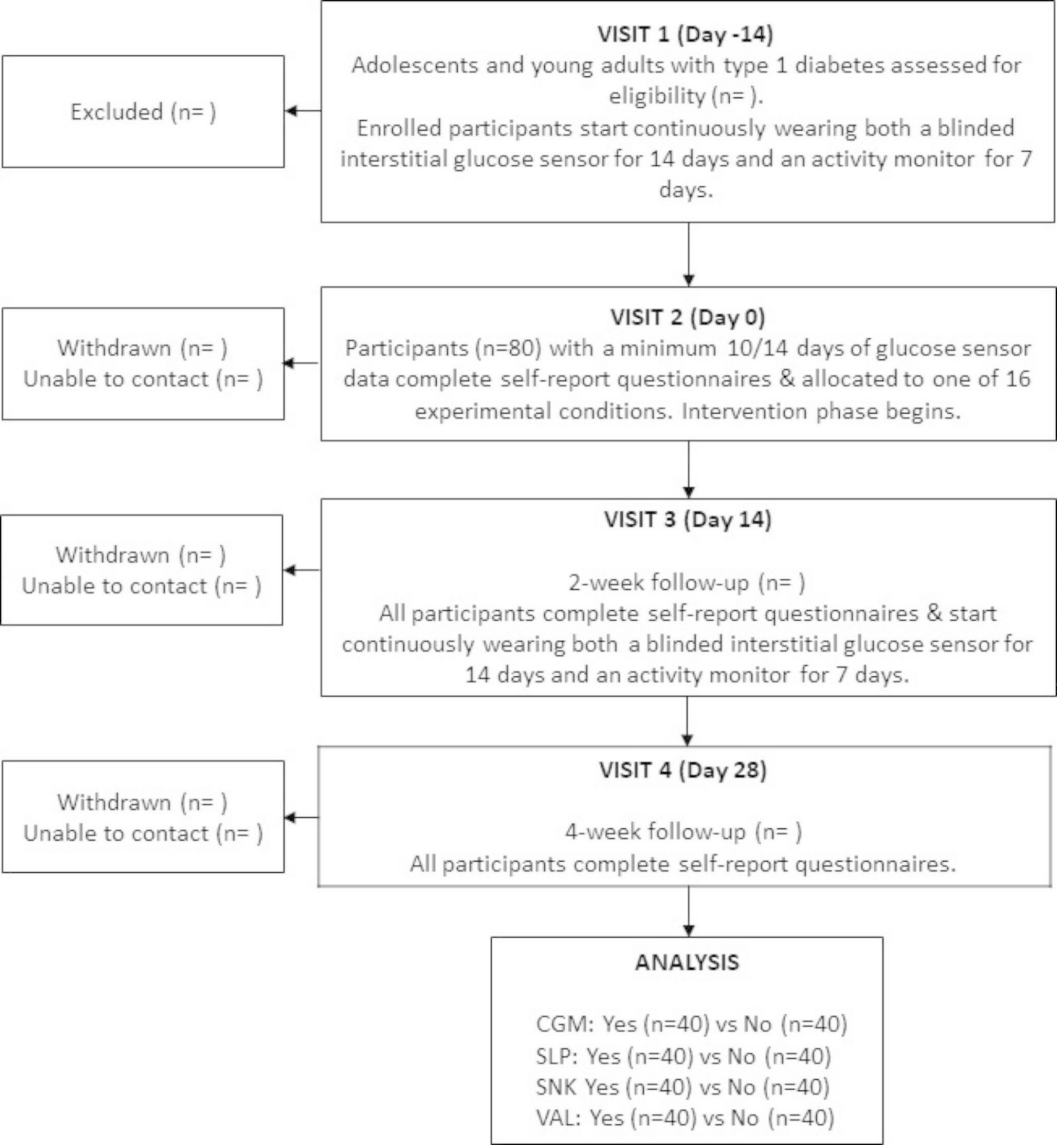
Flowchart of participant’s movement through the OPTIMISE study

As shown in Fig. 2, four study visits will occur over six weeks. All staff involved in data collection and delivering interventions will be trained in the study procedures by the principal investigator (S.E.S.). To promote participant retention, study visits will be conducted in participants’ preferred location (i.e., at home or a private research space) and up to three contact attempts will be made via email, text message and/or phone call to schedule study visits.

### Screening visit (day − 14)

Written informed consent will be obtained from participants aged 16–20 years and parents of those aged 13–15 years (written assent also obtained). Medical records will be reviewed to confirm the absence of exclusion criteria and mean of HbA1c results in the prior 6-months. Those who meet all inclusion, and no exclusion criteria, will be enrolled in the study and asked to apply a blinded CGM sensor and wear an ActiGraph initialised from the date of the screening visit.

### Baseline visit (day 0)

Research staff will check the blinded CGM snapshot report for a minimum of 10 days of data and screen for overnight hypoglycaemia. Participants will then be randomised to one of 16 experimental conditions (i.e., 0 to 4 of the self-care components) for the intervention phase and each allocated intervention will commence, as outlined in the supplementary file. Participants will be randomly allocated to experimental conditions using a computer-generated list of random numbers uploaded to the REDCap randomisation module by a biostatistician (J.J.H.). Each experimental condition represents a different treatment protocol. The experimental condition is concealed until research staff initiate randomisation in REDCap. Group allocation will be revealed after the participant has completed all baseline questionnaires. To reduce burden, those allocated to multiple interventions will begin the Dexcom® G6 CGM at the baseline visit and receive the remaining interventions at baseline or within seven days.

### Follow-up assessment (day 14)

All participants will repeat outcome assessments. CGM data and/or behaviour change goals will be reviewed on day 14 to identify potential opportunities to increase glucose TIR.

### Final assessment visit (day 28)

Participants will be re-assessed for study outcomes and provided with handouts of resources from components not received during the intervention phase. Participants will be offered a report of their glucose sensor data and advised to consult their diabetes team with questions. Participants will receive a $20 voucher as a token of appreciation.

### Safety monitoring

At the screening visit, participants who do not have a current hypoglycaemia management plan will be referred to their diabetes care team for advice. Anyone whose PHQ score is ≥ 15, DEPS-R score is ≥ 20 or self-reports recent episodes of diabetic ketoacidosis or severe hypoglycaemia at study visits will be referred to their usual diabetes care provider, general practitioner, or secondary care provider, as appropriate. Blinded CGM data will be reviewed and episodes where glucose values fall below 3.9 mmol/L overnight (10 pm to 7 am) will be reported to the participant’s diabetes care team, with their consent.

### Data management

To protect confidentiality, identifiable data (e.g., name, address, date of birth, date of diagnosis) will be collected with paper questionnaires or a password protected Excel document and stored securely at each site. All other study data will be collected and managed in REDCap.

### Statistical analysis

A biostatistician (J.J.H.) blinded to study group allocation will conduct all statistical analyses. Main and interaction effects will be estimated using a regression model with TIR as the outcome variable. Using effect coding (where each component is coded as 1 [received intervention] or -1 [did not receive intervention]), the main and interaction effects will be uncorrelated and therefore are similarly powered. The main effects inform the effectiveness of each component, while interaction effects inform how the components enhance or diminish the effects when together. Participants will be excluded from analyses if < 10 days of CGM data is captured and analyses using sleep data if < 3 nights of actigraphy data are obtained.

## Discussion

Adolescents and young adults with type 1 diabetes are required to perform multiple self-care tasks every day. The complexities associated with adhering to intensive self-management plans highlight the need for intervention studies to target direct and indirect factors that may support young people to achieving young people’s glycaemic goals. This research uses a pragmatic approach to develop an effective, robust, and scalable intervention to support young people with type 1 diabetes to increase their glucose TIR.

The strength of this research is the factorial experimental design that enables an estimation of the main effect of each component on TIR and the interactions between components.[8] This will result in an intervention that optimises both resource-use and health outcomes because only the most active combination of components will be included and less effective components will not. Once the optimised intervention is identified then its effectiveness can be assessed in a randomised controlled trial. This research will add to the evidence of effective diabetes self-care behaviour change strategies targeting a whole-person approach to the care of young people with type 1 diabetes.

## Electronic supplementary material

Below is the link to the electronic supplementary material.

Supplementary Material 1
