# Supplementary material for: The OPTIMISE study protocol: a multicentre optimisation trial comparing continuous glucose monitoring, snacking habits, sleep extension and values-guided self-care interventions to improve glucose time-in-range in young people (13–20 years) with type 1 diabetes
Source: J Diabetes Metab Disord. 2022 Aug 11;21(2):2023–33. doi: 10.1007/s40200-022-01089-x (PMC9672181; doi:10.1007/s40200-022-01089-x)
Supplement: Supplementary file 1 — Supplementary Material 1 [file 40200_2022_1089_MOESM1_ESM.docx]

3 June 2022

Professor Bagher Larijani

Editor-in-Chief, *Journal of Diabetes & Metabolic Disorders*

Dear Professor Larijani,

Please find attached the manuscript titled “The OPTIMISE study protocol: a multicentre optimisation trial comparing continuous glucose monitoring, snacking habits, sleep extension and values-guided self-care interventions to improve glucose time-in-range in young people (13-20 years) with type 1 diabetes” that we are submitting for consideration for publication in the *Journal of Diabetes & Metabolic Disorders*. We believe this manuscript fits well within the scope of the *Journal of Diabetes & Metabolic Disorders* and would be of interest to your readership.

This paper describes the study protocol for a 4-week intervention trial where 80 young people with type 1 diabetes will be randomised to one of 16 conditions in a factorial design using four intervention components: 1) real-time continuous glucose monitoring (CGM), 2) targeted snacking education, 3) individualised sleep extension, and 4) values-guided self-care goal setting. Baseline and post-intervention glucose time-in-range will be assessed with blinded CGM. Changes in self-care (snacking behaviours, sleep habits and duration, and psychosocial outcomes) will be assessed at baseline and post-intervention to determine if these interventions impacted behaviour change. Both main intervention and interaction effects will be estimated, and the optimised intervention will be evaluated to inform a future randomised controlled trial and guide the planning of effective clinical interventions in adolescents and young adults living with type 1 diabetes.

The clinical and scientific merit of this research protocol has undergone rigorous peer review by the primary funder, the Lottery Grants Board Health Research Grants of New Zealand, as well as the Department of Women’s and Children’s Health, University of Otago (Dunedin, New Zealand). The protocol has been reviewed by the Ngāi Tahu Research Consultation Committee and locality-based Māori Research Committees. The Northern A Health and Disability Ethics Committee gave ethical approval for the conduct of the OPTIMISE Study (20/NTA/127/AM03; Wellington, New Zealand).

This protocol has not been published or reviewed previously by any other journal. There are no conflicts of interest for any author. All authors have read and approved the final copy.

Thank you for your consideration of this manuscript. Please let me know if you have questions or require further information.

Dr Sara E. Styles

Department of Human Nutrition

University of Otago

PO Box 56

Dunedin, New Zealand

Email: sara.styles@otago.ac.nz
